# Quadriceps Tendon Allograft for Posterior Cruciate Ligament Reconstruction With and Without a Bone Block

**DOI:** 10.1002/atn2.70029

**Published:** 2026-05-14

**Authors:** Caleb Berta, Aqeel Nizar, Elizabeth Powell, Rucker Staggers, Brian Gilmer, Amit Momaya

**Affiliations:** ^1^ Department of Orthopaedic Surgery University of Alabama at Birmingham Birmingham Alabama U.S.A.; ^2^ Reno School of Medicine University of Nevada Reno Nevada U.S.A.; ^3^ Mammoth Orthopedic Institute Mammoth Hospital Mammoth Lakes California U.S.A.

## Abstract

Graft options for posterior cruciate ligament reconstruction include both autografts and allografts, with hamstring autografts and Achilles tendon allografts being the most common options. Although not as utilized, quadriceps tendon allografts have advantages similar to those that have driven the use of quadriceps autografts in knee reconstruction, such as favorable biomechanical properties and increased cross‐sectional area. This article describes the use of quadriceps tendon allograft with bone block and without bone block for posterior cruciate ligament reconstruction. Surgical techniques, fixation methods, and clinical indications of each graft are discussed, as well as the pearls and pitfalls of each technique.

## Graphical abstract

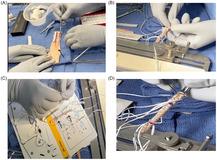

**VIDEO 1:** This video shows a technique for quadriceps tendon (QT) allograft reconstruction with and without a bone block. We begin with an all‐soft tissue graft where a double bundle technique is shown. Sizing of the graft for each bundle is showed. Following graft preparation, whipstitching is done, followed by tensioning of the graft for storage. The technique for the bone block allograft is then presented. For this technique, graft sizing and preparation are shown. Preparation of the soft tissue side of this graft is done similarly to the anterolateral bundle of the all‐soft tissue graft. For the preparation of the bone block, 2 holes are drilled into the bone block, and a suture is passed through each hole. For the allograft with a bone block, drilling of the right knee's tibial and femoral tunnels is shown. The tibial side involves forming a socket with a FlipCutter. The femoral side involves full tunnel drilling. The graft is then passed through the femoral tunnel into the tibial side. The video concludes by showing fixation using the interference screw technique on the femoral side. Video content can be viewed at https://doi.org/10.1002/atn2.70029.

A large number of autografts and allografts exist for posterior cruciate ligament (PCL) reconstruction. These grafts use the following tendons: Achilles, tibialis anterior or posterior, peroneus longus, bone–patellar–tendon–bone, hamstrings, and quadriceps tendon (QT).^1^ Although the Achilles tendon allograft is most often used in PCL reconstruction, the QT has seen increased use in various ligamentous reconstructive procedures, including PCL reconstruction.^1^, ^2^

Studies have shown that the QT allograft is biomechanically equivalent to an Achilles allograft, including a similar cross‐sectional area and load to failure.^3^, ^4^ Other literature has found no significant differences in clinical or radiologic outcomes between Achilles tendon and QT allografts for PCL reconstruction.^5^ These findings support the QT as an alternative to Achilles for PCL reconstruction, and gives surgeons a broader ranges of grafts to choose from.

In this article, we describe surgical techniques, fixation methods, and clinical indications for the use of QT allograft with and without bone block, as well as the pearls and pitfalls of each technique (Table 1).

**TABLE 1:** Pearls and Pitfalls of Quadriceps Tendon Bone versus Quadriceps Tendon Soft Tissue

**Aspect**	**Pearls**	**Pitfalls**
Graft Type	QT allograft offers high biomechanical strength and versatility and can be used with (QT‐B) or without bone block (QT‐S).	No consensus on preferred graft type in PCL reconstruction.
QT‐S Graft Preparation	Uses tapered ends and FiberTag tightrope with cortical button for secure fixation. Pretension and compress in an undersized graft tube to ease passage.	Graft swelling can complicate passage through the “killer turn.”
QT‐B Graft Preparation	Bone block offers potential for bone‐to‐bone healing and interference screw fixation on femoral side adds stability.	Improper bone block sizing can complicate “killer turn” passage.
Tunnel Drilling	QT‐S allows for an all‐inside technique which minimizes cortical disruption.	QT‐B requires more extensive femoral tunnel drilling.
Fixation	QT‐S uses suspensory fixation on both sides whereas QT‐B uses suspensory fixation on the tibial side and interference screw fixation on the femoral side.	Uses of suspensory fixation can lead to tunnel widening due to graft micromotion within the tunnel.
Sizing and Tensioning	Use 9‐10 mm diameter depending on patient size; pretension to 20 lbs. improves graft handling.	Oversized grafts may lead to loss of motion, arthrofibrosis, and cyclops lesions.
Clinical Consideration	QT allograft adds to the viable options for PCL reconstruction when limited allografts are available.	Quality of graft varies based on irradiation and sterilization techniques.

PCL, posterior cruciate ligament; QT, quadriceps tendon.

## SURGICAL TECHNIQUE

Surgical techniques for graft preparation are presented in Video 1.

### Graft Preparation

#### Quadriceps Tendon Allograft All Soft Tissue (QT‐S)

##### Graft Dimensions

The graft that functions as the anterolateral (AL) bundle measures 90 mm in length with a diameter of approximately 10 mm. The graft that functions as the posteromedial (PM) bundle measures 70 mm in length with a diameter of 6‐7 mm.

#### Graft Preparation

##### Allograft

**FIGURE 1:**
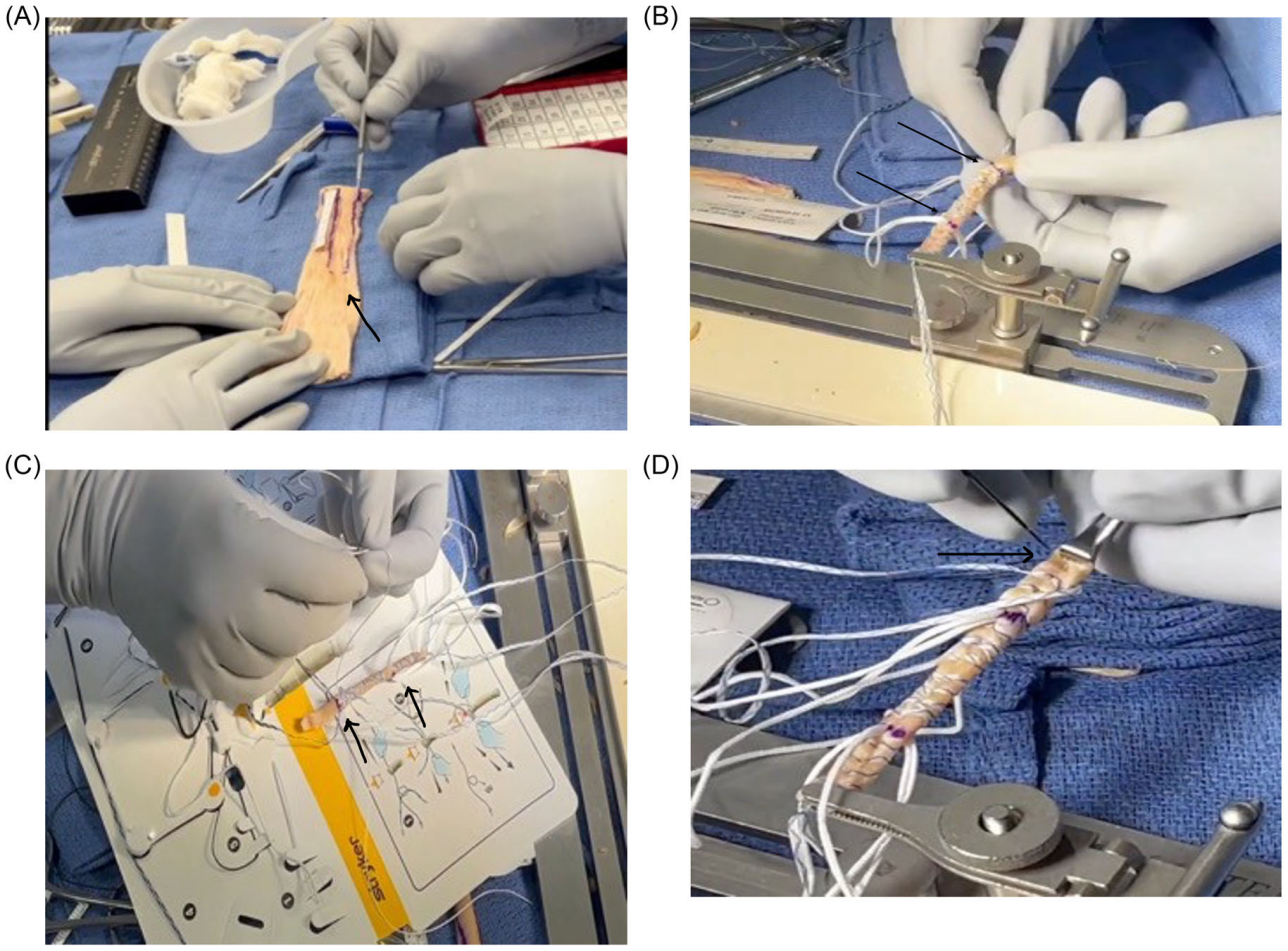
(A) Shows the quadriceps tendon allograft (arrow) being templated and carefully incised with a 10‐blade scalpel with sharp borders. (B) Shows the quadriceps tendon allograft being whipstitched using 1.4 mm looped suture tapes up to the markings (arrows). (C) Shows the ProCinch Open Loop Cortical Button System being applied to the ends of each quadriceps tendon allograft at the blue markings (arrows). (D) Shows the looped suture tape being whipstiched (arrow) past the ProCinch device to the ends of the quadriceps tendon allograft.

The appropriate graft size is marked out for both the AL and PM bundles. The thicker medial aspect of the allograft is used for the larger AL bundle, and the thinner lateral aspect of the graft is used for the smaller PM bundle. The tendon is carefully incised with a 10‐blade scalpel with sharp borders (Figure 1A). Each end is tapered using Iris or Metzenbaum scissors to help facilitate graft passage. The graft is marked 30 mm from the tibial end and 20 mm from the femoral end to guide tunnel positioning.

##### PM Bundle

First, the entire graft is whipstiched to mitigate the graft from “accordioning” during graft passage. To do this, two 1.4 mm looped suture tapes (Stryker, Kalamazoo, MI) are whip stitched, starting from the middle and then working up to the previous markings on the graft until the next step is completed (Figure 1B). The ProCinch Open Loop Cortical Button System (Stryker, Kalamazoo, MI) is applied to each graft at the optimal position (30 mm on the tibia and 20 mm on the femur) by passing suture across the graft and then converting the button to a closed adjustable loop according to the manufacturer's specified technique (Figure 1C). The whipstitch is continued to the ends of the graft and tied (Figure 1D).

##### AL Bundle

Two Procinch Open Loop Cortical Button Systems are applied as previously described. However, only the ends are whip stitched using a similar 1.4 mm looped suture.

##### Back Table Holding

The graft is pretensioned to 20 pounds and covered with a Raytec soaked in 5 mg/mL vancomycin solution (1 g in 200 mL solution) until the time of implantation.

#### Tunnel Drilling

##### Tibial Tunnel

**FIGURE 2:**
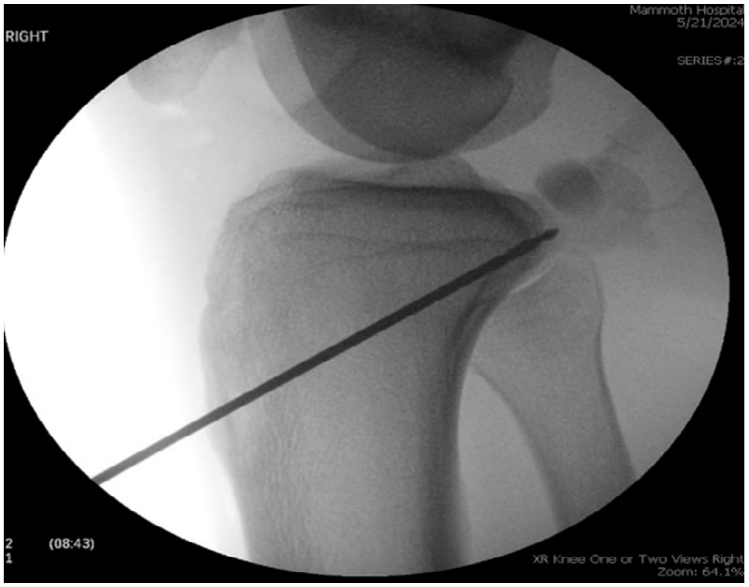
Shows a fluoroscopic view of the right knee. A guide pin is placed under direct visualization and fluoroscopy to confirm the appropriate position for the tibial tunnel.

A PCL tibial guide (Arthrex, Naples, FL) is set to 60° and introduced into the knee, hooking around the posterior tibia above the champagne glass drop off. A guide pin is placed under direct visualization, and fluoroscopy confirms the appropriate position (Figure 2). A solid reamer is used to make an 11.5 mm tunnel, using the PCL guide to push back the soft tissues in the posterior knee. Three FiberStick (Arthrex, Naples, FL) sutures are passed up the tibial tunnel and stored in the anteromedial portal for later use to pass the grafts.

##### Femoral Tunnel

**FIGURE 3:**
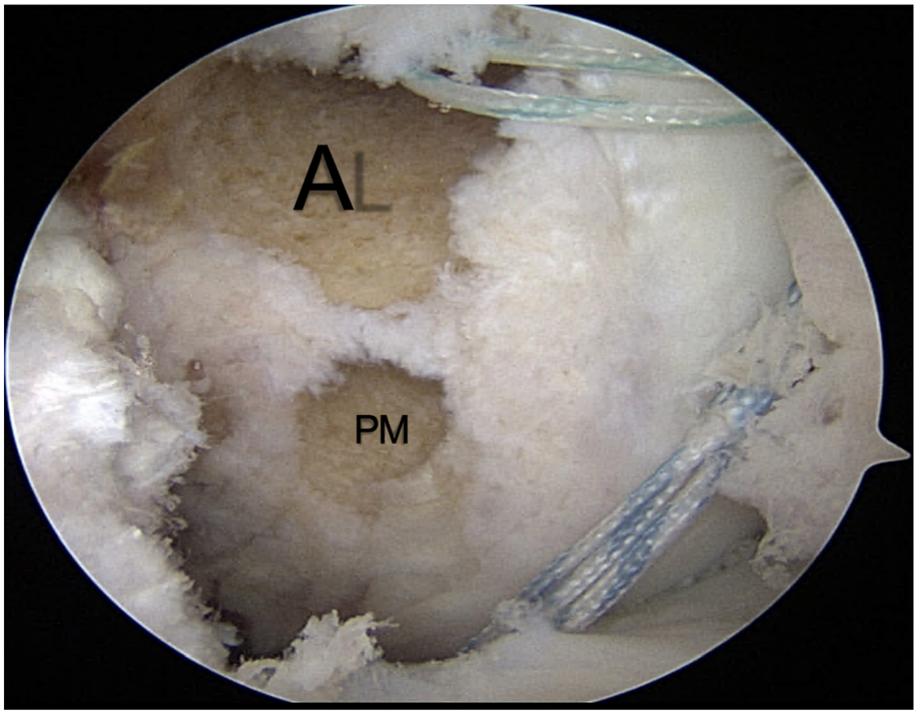
Shows an arthroscopic view of the right knee with a 30‐degree scope. The appropriate tunnel position at the anatomic origin of each PCL bundle are shown anterolateral (AL) and posteromedial (PM).

A designated guide (Arthrex, Naples, FL) is placed along the lateral aspect of the medial femoral condyle at the anatomic origin of each PCL bundle, ensuring that the guide is just proximal to the articular margin (Figure 3). The PM tunnel is reamed first, followed by the AL tunnel. Tunnels are reamed line‐to‐line, meaning a graft with a diameter of 6 mm gets a 6 mm tunnel and a graft with a diameter of 10 mm gets a 10 mm tunnel. This is done using a 3.5 mm guide pin and FlipCutter (Arthrex, Naples, FL). Three FiberStick sutures are placed as passing sutures. The PM bundle uses a single passing stitch while the AL bundle uses two passing sutures, one of which will be designated for internal brace augmentation.

#### Graft Passage

The PM bundle is shuttled through the anteromedial portal into the tibial side first and is pulled retrograde into the appropriate femoral tunnel. An 8 × 12 mm femoral button is applied. The FiberTape internal brace (Arthrex, Naples, FL) is folded in half, and the looped end is passed through the AL femoral tunnel and clamped. The two tails of the internal brace are then passed into the tibial tunnel. The AL bundle graft is drawn into the tibia, while maintaining tension on the internal brace and PM graft to allow smooth graft passage. An 8 × 12 mm femoral button is secured first to the looped end of the internal brace, followed by attachment of the AL graft suspensory fixation, and then the construct is tensioned. Finally, a large 20 mm tibial button is applied to both graft suspensory loops (excluding the internal brace), and each graft is tensioned back to the tibia. The AL bundle is tightened in 90° flexion and the PM bundle in 20° of flexion. The internal brace is independently fixed at 90° of flexion to an anchor on the anteromedial tibia with a freer placed beneath to avoid overtensioning. After which, the knee is cycled and retensioned to reduce creep.

### QT Allograft with Bone Block (QT‐B)

#### Graft Dimensions

**FIGURE 4:**
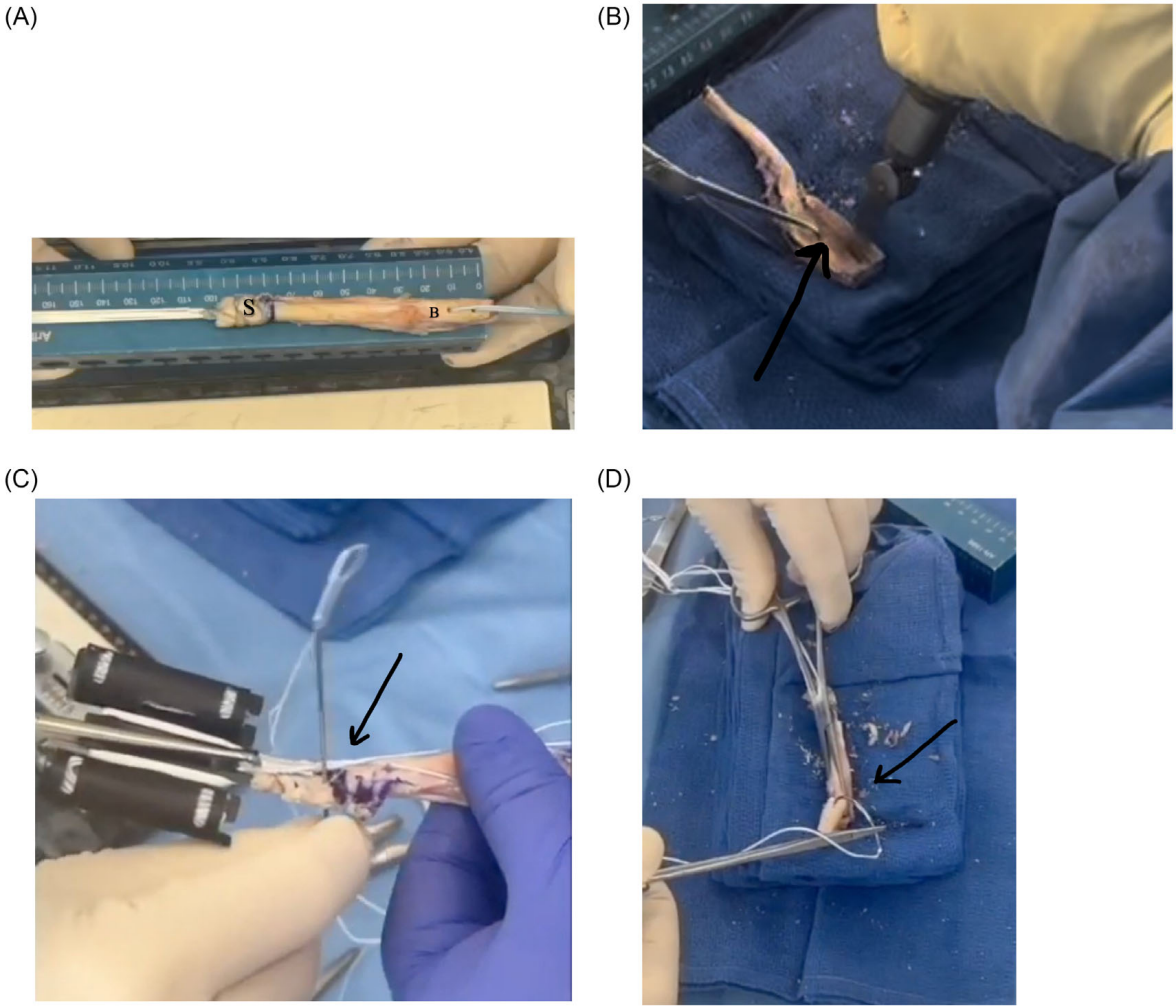
(A) Shows the quadriceps tendon allograft with a bone block. Whipstitching has been done on the soft tissue end (S), and 2‐0 FiberWire suture has been put through the bone block end (B). The graft measures approximately 9‐10 mm in diameter with a length of approximately 95 mm. (B) Shows use of a saw blade to size the patellar bone block (arrow) to the appropriate size. (C) Shows whipstitching (arrow) of the soft‐tissue end of the quadriceps tendon graft with bone block. (D) Shows placement of a 2‐0 FiberWire (arrow) into the bone block end of the graft.

The graft measures approximately 9‐10 mm in diameter and 90‐95 mm in length, with 25‐30 mm coming from the bone block harvested from the superior patella (Figure 4A).

#### Graft Preparation

##### Allograft

Since the allograft is large, the appropriate graft size is marked out using the GraftPro Preparation station (Arthrex, Naples, FL). The soft tissue end of the graft is carefully incised with a fresh 15‐blade scalpel. A saw blade is used to size the bone to approximately 25‐30 mm in length and 9‐10 mm in diameter, followed by tapering with a rongeur to facilitate smooth tunnel passage (Figure 4B).

##### Tibial End

The soft tissue end of the graft was prepared with whipstitching using FiberTag (Arthrex, Naples, FL) in similar fashion to the AL side of the QT‐S graft (Figure 4C).

##### Femoral End

The bone end of the graft is drilled and two #2‐0 FiberWire (Arthrex, Naples, FL) are passed through the bone with needle drivers (Figure 4D).

#### Tunnel Drilling

##### Tibial Side

**FIGURE 5:**
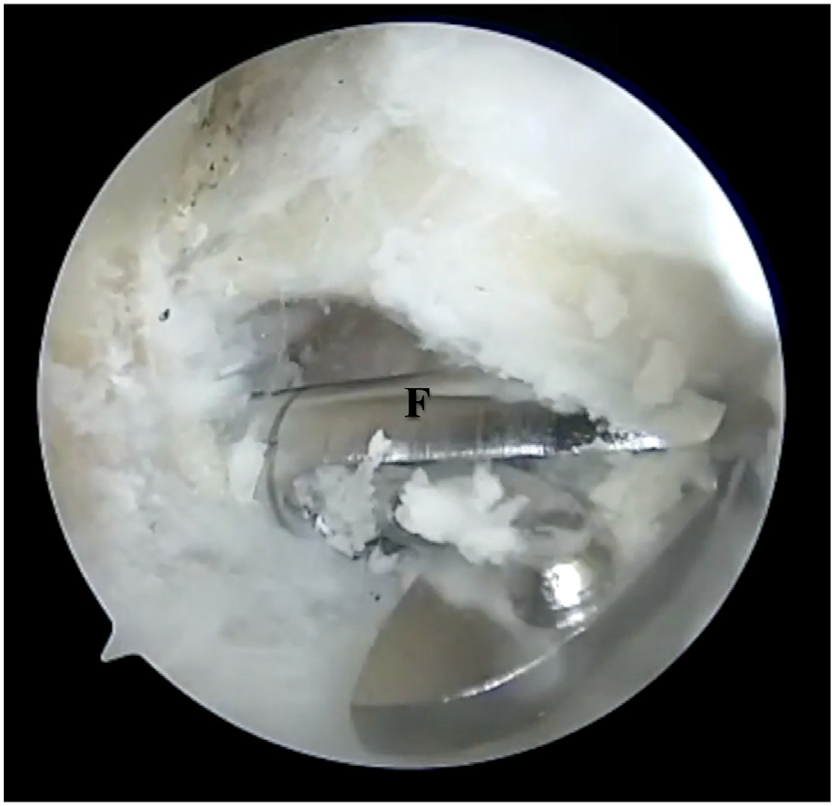
Shows an arthroscopic view of the right knee with a 30‐degree scope. A socket is being drilled on the tibia using a FlipCutter.

A 10 mm socket is drilled on the tibial side (Figure 5) using the FlipCutter (Arthrex, Naples, FL).

##### Femoral Tunnel

**FIGURE 6:**
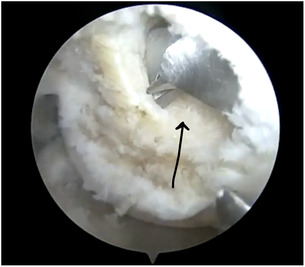
Shows an arthroscopic view of the right knee with a 30‐degree scope. A full tunnel is being drilled on the femoral side (arrow).

A full 10 mm tunnel is drilled on the femoral side (Figure 6).

#### Graft Passage

**FIGURE 7:**
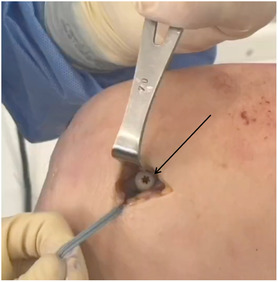
Shows fixation of the bone plug with an interference screw (arrow) on the femoral side of the graft.

The graft is passed through the knee from the femoral side to the tibial side using a suture retriever to pull the graft through from the soft tissue side of the graft. The FiberWire are used to hold tension on the graft during passage. Suspensory fixation of choice is used on the soft tissue tibial side, and interference screws are used on the bone block on the femoral side (Figure 7).

## DISCUSSION

This technique describes the use of QT allograft with and without a bone plug for PCL reconstruction. The QT autograft has emerged as a reliable and versatile option for ligamentous reconstruction due to its favorable biomechanical and anatomical properties and has several distinct advantages in comparison to other grafts. Its thickness and width allow for individualized graft sizing, making it suitable for both single‐bundle and double‐bundle reconstruction techniques. The QT's length enables the use of longer grafts, which is particularly beneficial in PCL reconstruction, where grafts must cover a greater intra‐articular distance in comparison to ACL reconstruction.^6^ The length of the graft allows for adequate intra‐articular coverage and secure fixation within the tunnels, regardless of patient size.

Additionally, the QT can be harvested with or without a bone plug, providing further flexibility in surgical approach.^7^ Although tunnel widening has not been extensively studied in PCL reconstruction, its significance in ACL surgery suggests a similar relevance in PCL reconstruction.^8^ Tunnel widening can complicate revision procedures, often requiring a staged revision, and both graft choice and fixation method influence its occurrence.^9^ QT‐B grafts with interference screw fixation may reduce widening compared with QT‐S grafts using suspensory fixation, with selection guided by surgeon preference and patient‐specific factors.^10^, ^11^

Allografts are an attractive alternative to autograft that allows for shorter surgical time and less donor site morbidity.^12^ In PCL reconstruction, the extensor mechanism provides an important protective force on the PCL; therefore, QT allograft is preferable to autograft in order to preserve this function.^13^, ^14^, ^15^ In addition, a systematic review by Migliorini et al. reported no significant differences between autografts and allografts in PCL reconstruction for Lysholm score, range of motion, Tegner scale, IKDC score, arthrometer‐measured laxity, drawer test results, or Telos stress radiography findings.^16^

The QT allograft, which can be utilized with or without a bone plug, offers a clinically effective option for PCL reconstruction, with graft selection best guided by patient‐specific factors and surgeon preference.

## DISCLOSURES

The authors (B.G., A.M.) declare the following financial interests/personal relationships which may be considered as potential competing interests: B.G. reports a relationship with Miach Orthopaedics that includes consulting or advisory, reports a relationship with AlloSource that includes consulting or advisory, reports a relationship with CONMED that includes consulting or advisory, reports a relationship with Vertex Pharmaceuticals that includes speaking and lecture fees, and reports a relationship with Arthrex that includes consulting or advisory. B.G., given his role in the Editorial Board of *Arthroscopy*, had no involvement in the peer review of this article and had no access to information regarding its peer review. Full responsibility for the editorial process for this article was delegated to another journal editor. A.M. reports financial support provided by AlloSource and reports a relationship with CONMED that includes consulting or advisory. A.M., given his role in the Editorial Board of *Arthroscopy*, had no involvement in the peer review of this article and had no access to information regarding its peer review. Full responsibility for the editorial process for this article was delegated to another journal editor. The other authors (C.B., A.N., E.P., R.S.) declare that they have no known competing financial interests or personal relationships that could have appeared to influence the work reported in this article.
